# Selection and plasticity both account for interannual variation in life‐history phenology in an annual prairie legume

**DOI:** 10.1002/ece3.5953

**Published:** 2020-01-10

**Authors:** Amber R. Nashoba, Thomas J. Y. Kono

**Affiliations:** ^1^ Department of Biological Sciences University of Alaska Anchorage Alaska; ^2^ Minnesota Supercomputing Institute Minneapolis Minnesota

**Keywords:** Aster models, *Chamaecrista fasciculata*, effect of environment, life‐history

## Abstract

As the environment changes, so too must plant communities and populations if they are to persist. Life‐history transitions and their timing are often the traits that are most responsive to changing environmental conditions. To compare the contributions of plasticity and natural selective response to variation in germination and flowering phenology, we performed a quantitative genetic study of phenotypic selection on *Chamaecrista fasciculata* (Fabaceae) across two consecutive years in a restored tallgrass prairie. The earliest dates of germination and flowering were recorded for two parental cohorts and one progeny cohort in an experimental garden. Environmental differences between years were the largest contributors to phenological variation in this population. In addition, there was substantial heritability for flowering time and statistically significant selection for advancement of flowering. Comparison between a progeny cohort and its preselection parental cohort indicated a change in mean flowering time consistent with the direction of selection. Selection on germination time was weaker than that on flowering time, while environmental effects on germination time were stronger. The response to selection on flowering time was detectable when accounting for the effect of the environment on phenotypic differences, highlighting the importance of controlling for year‐to‐year environmental variation in quantitative genetic studies.

## Graphical abstract

Our study is a phenotypic selection analysis of total lifetime fitness in a natural selective prairie environment. We compare the predicted and observed phenotypic response, taking care to account for the effect of year‐to‐year variation. We observe a change in mean flowering time that is consistent with the direction of selection when accounting for the effect of the environment, highlighting the importance of controlling for temporal variation in multiyear quantitative genetic studies.

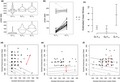

## 1 INTRODUCTION

Recent climate change in the northern hemisphere has resulted in a northward shift in environmental conditions (Davis & Shaw, 2001; Parmesan & Yohe, 2003; Shaw & Etterson, 2012). Optimal environments for a given population may now be at higher latitudes than in the recent past (Bennington et al., 2012). Future climate change is projected to occur more rapidly than past climate change; the 2013 Intergovernmental Panel on Climate Change (IPCC) report projects an increase in central North American temperatures of 1.0–1.5 degrees Celsius (Annex 1), and a decrease in moisture availability of 1%–2% (Chapter 11) from 2016 to 2035 (IPCC, 2013). Projected climate change may threaten the persistence of plant populations, as they are primarily sessile and generally well adapted to their historic ranges (Turesson, 1922).

Among the most immediate and visible responses to climate change are shifts in phenology (Parmesan & Yohe, 2003). In a 30‐year study of more than 500 European plant species from 21 countries, leaf‐out and reproductive phenology advanced significantly in 30% of records (Menzel et al., 2006). A Mediterranean study during approximately the same period found evidence for advancing phenology in both plant and animal species, with insects advancing faster than plants (Gordo & Sanz, 2005). Reproductive phenology in vertebrate populations has also shifted. Historical records covering 10 to 69 years show that for every one‐day advancement in ice‐out date, walleye (*Sander vitreus*) spawning has advanced 0.5–1.0 days in Minnesota (Schneider, Newman, Card, Weisberg, & Pereira, 2010). Observational studies such as these can be used to inform qualitative predictions of how species will respond to future change. However, an experimental approach that includes the direct phenotypic comparison of parent and offspring generations has the power to distinguish a genetically based response to selection from phenotypic plasticity (Franks & Weis, 2008).

Heritabilities of, and genetic correlations among traits under selection serve as a theoretical framework for the empirical study of adaptation (Falconer & Mackay, 1996). For example, the response to selection depends on the heritabilities of individual traits and their effect on fitness, and genetic correlations between traits may either enhance or constrain the realized response to selection (Etterson & Shaw, 2001; Kelly, 1993; Réale, Berteaux, McAdam, & Boutin, 2003). In *Chamaecrista fasciculata*, Kelly (1993) detected significant heritability in the phenology of life‐history traits while Etterson and Shaw (2001) reported that genetic correlations between traits were expected to impede the response to selection. By studying a population with a known pedigree, these experiments provided information on the genetic variation of traits under selection.

The nature of selection is reflected in the relationship between Darwinian fitness and other traits. These relationships, or modes of selection, are evident from a population's distribution of individual fitness, in which the highest fitness values are observed to coincide with nonfitness trait values that are intermediate, extreme, or highest/lowest (respectively, stabilizing, disruptive, or directional selection [Endler, 1986]). Explicit study of how traits covary with fitness can reveal the mode of selection acting on those traits. One approach to estimating the relationships between fitness and the phenotypic values of traits of interest was developed by Lande and Arnold (1983). Their approach predicts fitness as a function of predictor trait values, which allows inference of trait values that are associated with the highest fitness in a population (Schwaegerle & Levin, 1991; Walsh & Blows, 2009). One complication with modeling fitness is that it depends on multiple life‐history stages, such as germination and the juvenile stages prior to reproduction. Fitness and life‐history stages must be considered jointly, which is possible through the use of Aster models (Geyer, Wagenius, & Shaw, 2007).

The mode and strength of selection acting on a population are temporally variable, and predictions of a population's long‐term response to selection require observations of that population across multiple generations. One way to obtain such observations in a natural environment is by examining spatial variation in environmental conditions as an approximation for anticipated future climatic conditions (Shaw & Etterson, 2012). Another approach would be to observe a population in the same geographic setting at multiple time points, as is done in the current study.

For this study, *C. fasciculata* seeds were sampled from a Minnesota prairie on the bluffs of the Mississippi River and used to generate a pedigreed population. Crosses were performed both in the field and among greenhouse‐raised plants. Seeds of known pedigree were then planted into a restored prairie experimental garden. From these individuals, phenology and fitness were assessed for three cohorts: two first generations (grown in 2013 and 2014) plus one offspring cohort (2014). We seek to address three central questions in this study. First, how does life‐history phenology (germination date and the date of first flower) change in response to environmental differences between years? Second, what is the mode of selection acting on these phenological traits, and what are the realized responses to selection in the offspring generation? Third, what are the additive genetic variances for germination time and flowering time and are these parameters correlated? To address these questions, we used Aster models (Geyer et al., 2007) to examine selection and the phenological response to selection on germination date and flowering date, and we used Quercus software (Shaw & Shaw, 1994 and 2016) to estimate genetic variances and correlations. Aster models are well suited to these analyses because they account for the contributions of individual life‐history stages to lifetime fitness. This study includes three major ways to assess the effect of the environment on phenotypic variation. The first is direct estimation of heritability in multiple environments. The second is observing phenotypic plasticity by comparing the relative trait values of pedigreed families across multiple environments. Finally, the selective environment was measured over multiple years. While each of these aspects of the effect of the environment is not completely independent, they each represent different ways to assess how phenotypes change in relation to environmental conditions.

## 2 MATERIALS AND METHODS

### 2.1 Study species

*Chamaecrista fasciculata* is an annual legume that occurs in disturbed sites such as roadsides and regularly burned prairies. Its native range is in North America from the East Coast to just west of the Mississippi River, spanning latitudes from Central Minnesota to Northern Mexico. This species has perfect flowers, enabling an individual to serve as either maternal or paternal parent in crosses conducted to generate a pedigreed population. Its annual life cycle makes *C. fasciculata* suitable for estimating lifetime fitness, as the total reproductive output of a generation of individuals can be measured in a single year. In Minnesota, germination generally begins in May and continues through June (Nashoba, personal observation). Plants in this region at the northern range edge tend to transition to reproductive maturity starting in July and continue to flower until killed by frost.

### 2.2 Experimental population development

All source material for the experimental population was derived from seeds collected over approximately 136 acres of the 237‐acre Grey Cloud Dunes (GCD) Scientific and Natural Area in Cottage Grove, Minnesota (44°47′26.6″N 92°57′30.2″W). This prairie site on the bluffs of the Mississippi River has sand‐gravel soil. In addition to *C. fasciculata*, the plant community at GCD includes big bluestem (*Andropogon gerardii*), little bluestem (*Schizachyrium scoparium*), sandbur (*Cenchrus longispinus*), *Liatris*, and *Dalea*.

**Figure 1:**
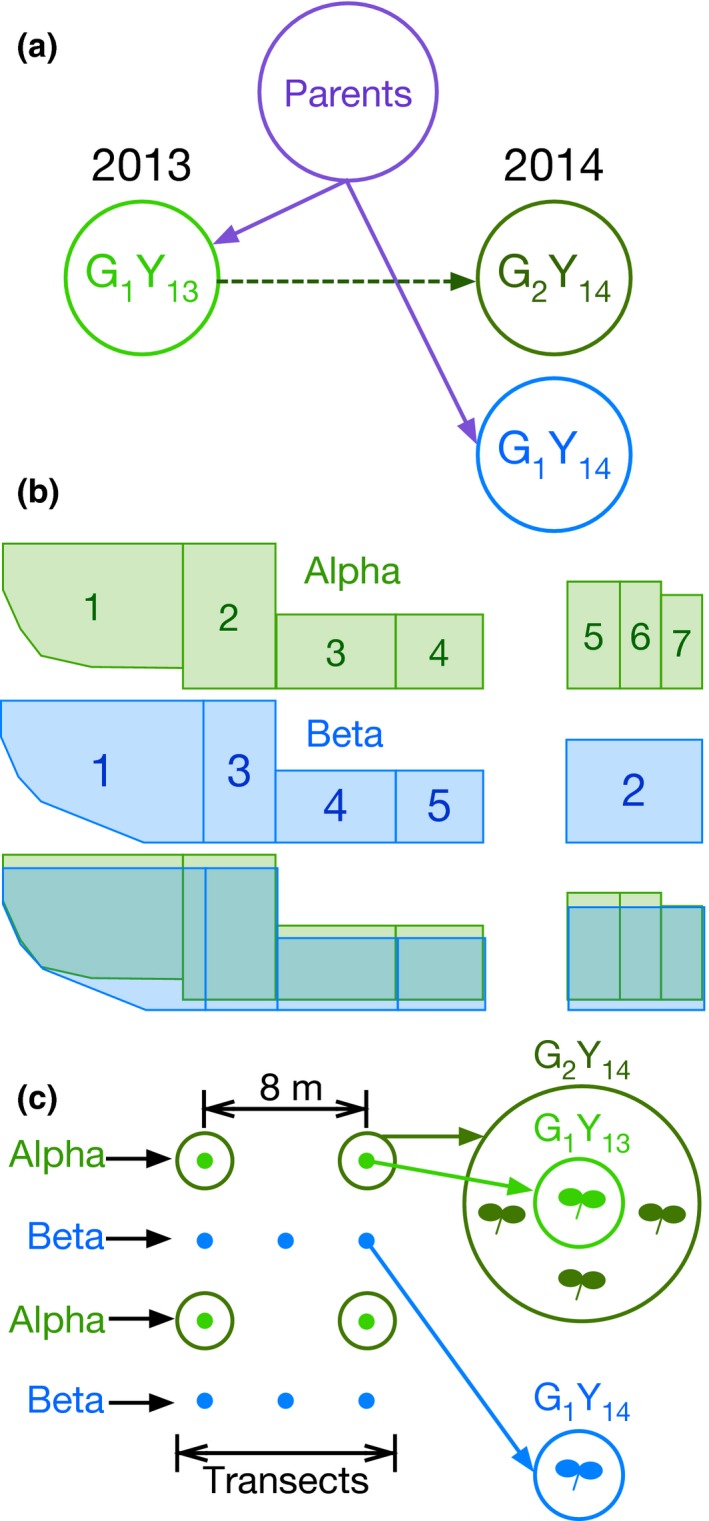
(a) Schematic of the relationships among the three cohorts in this study. G_1_Y_13_ and G_1_Y_14_ were derived from controlled crosses, and G_2_Y_14_ was produced from natural outcrossing among G_1_Y_13_ individuals. (b) Diagram showing the layout of the blocks within the experimental field site. The 2013 growth year is shown in green, and the 2014 growth year is shown in blue. (c) Within blocks, planting locations were organized along transects. Alpha group transects (green points and circles) were spaced eight meters apart, with planting locations every eight meters. Beta group transects (blue points) were interleaved with alpha group transects and had planting locations every 4 m

For quantitative traits, estimating the genetic contribution to phenotypic variation requires a population of known pedigree. A pedigreed population of *C. fasciculata* was generated in the scheme outlined in Figure 1a. High mortality and low pollination success necessitated three crossing efforts (Table S1). In each effort, individual seedlings were assigned to crossing groups at random. The first round of crossing took place in a greenhouse at the University of Minnesota Plant Growth Facility, using seeds harvested from GCD in fall of 2011. These seeds were obtained from the up to four mature fruits that were collected from each of 200 maternal individuals. Sampled individuals were separated by a minimum of five meters along parallel transects established three meters apart. In early 2012, the collected seeds were germinated, reared, and the 165 surviving individuals were hand‐pollinated using a reciprocal factorial crossing design. Under this design, individuals in each crossing group served as both pollen donors and pollen recipients; this did not include self‐pollinations. The second crossing effort took place at the source population site during the 2012 growing season and utilized a paternal half‐sibling nested design. In this method, each crossing group consists of one individual serving as the pollen donor (sire) and approximately three individuals as pollen recipients (dams). Beginning in mid‐May, 285 seedlings were monitored at GCD. Initially, 200 individuals were selected at random as seedlings and assigned to crossing groups. If a selected plant died before flowering, a replacement individual was chosen at random. To ensure that designated pollen recipients were not pollinated prior to manual pollination and to ensure that pollen from pollen donor flowers was not exhausted, 15 mm sections of biodegradable drinking straws were placed over flower buds prior to blooming.

From the two initial crossing efforts, 20% of seed from each full‐sibling family with more than five seeds was reserved to act as pollen donors and pollen recipients in the third crossing effort. In the greenhouse in early 2013, 428 reserved seeds from 102 full‐sibling families were reared and pollinated using a reciprocal factorial crossing design, excluding self‐fertilization as in the first effort.

The seeds resulting from the first two crossing efforts made up the first‐planted pedigreed cohort (G_1_Y_13_) and the seeds from the third effort comprised the second pedigreed cohort (G_1_Y_14_). These controlled crosses formed the experimental population used in this study (Table S1). These generation 1 (G_1_) cohorts are not genetically identical; they are neither clones nor inbred lines. However, because the parents of both G_1_ cohorts were representative of the study population and mated at random, the genotypic frequencies of the G_1_ cohorts are expected to be equal to those of their parents and, thus, the frequencies of the two G_1_ cohorts will be approximately equal (Hardy, 1908). However, we acknowledge that differences likely exist between G_1_Y_13_ and G_1_Y_14_ due to the sampling effects during controlled crosses. While it is possible that maternal effects will contribute to phenotypic variance (e.g., between the offspring of crosses conducted in the greenhouse and crosses conducted in the field), additive genetic variance was estimated using paternal half‐sibling relationships (Falconer & Mackay, 1996). Additionally, the effect of crossing effort was not significant in our Aster model analysis (see Section 3).

### 2.3 Experimental garden

Pedigreed seeds were planted in an experimental garden in Shakopee, Minnesota (44°46′13.7″N 93°26′46.9″W) on land that is owned and managed by the Shakopee Mdewakanton Sioux Community. The garden occurs 38.5 km west of the source population site on sandy loam soil and lies in a three‐hectare unit of restored prairie. Restoration of the experimental site to tall‐grass prairie from agricultural usage occurred in 2008. The diverse plant community at this site includes the dominant grass species Canada rye (*Elymus canadensis*), big bluestem (*Andropogon gerardii*), and *Sorghastrum nutans*. Forbs and legumes are also well established and include species of *Aster*, *Solidago*, *Monarda*, *Asclepias*, *Dalea*, *and Astragalus*. The site was first burned in the third week of November 2012; the first group of pedigreed seed (G_1_Y_13_) was planted into the experimental garden approximately one week later. Prior to planting the second group of pedigreed seed (G_1_Y_14_) in the following year, all planting locations were mowed. Planting in both years commenced following the first hard frost to ensure that individuals would overwinter as seed and continued until all experimental individuals had been planted.

To enable comparisons between generations and growth years, a total of three cohorts were evaluated over a period of two growing years. The two G_1_ cohorts were designated Alpha group (G_1_Y_13_) and Beta group (G_1_Y_14_). Comparisons within the Alpha group would conflate the effect of the environment and response to selection; comparisons between Alpha and Beta either allow examination of the effect of the environment or the genetic response o selection. Individuals of the G_1_Y_13_ cohort were allowed to outcross naturally, and the resulting offspring established the second‐generation G_2_Y_14_ cohort and belong to the Alpha group. In 2014, first‐generation Beta group cohort G_1_Y_14_ and second‐generation Alpha cohort G_2_Y_14_ were grown contemporaneously (Figure 1a).

The G_1_Y_13_ cohort (Alpha group) was planted into a randomized block design with seven blocks from 28 November through 02 December 2012. The G_1_Y_14_ group of pedigreed seeds (Beta group) was planted into five blocks from 30 November 2013 through 11 January 2014 (Figure 1b). Groups of up to five full‐sibling seeds were planted in clusters at locations organized along transects with each block. The mean number of seeds planted per location to establish the G_1_Y_13_ pedigreed Alpha cohort was 4.4 seeds, and the mean number of seeds planted per location for G_1_Y_14_ pedigreed Beta cohort was 4.9 seeds. G_1_Y_13_ individuals were allowed to naturally outcross to form the second‐generation G_2_Y_14_ cohort_._ This G_2_ cohort comprised individuals that were naturally dispersed as well as those that were harvested from randomly sampled parental (G_1_Y_13_) fruits. Seeds from these fruits were planted at toothpicks adjacent to their maternal plant.

Alpha group transects were spaced eight meters apart, with planting locations occurring every four meters along the transect (Figure 1c). Beta group transects were established as alternating between Alpha group transects, with planting locations spaced every four meters (Figure 1c). In total, 2,370 G_1_Y_13_ seeds were planted at 533 planting locations; 19 of these locations were planted with a total of 90 seeds of unresolved pedigree. G_1_Y_14_ was planted with 5,193 seeds at 1,054 planting locations, and 6,842 G_2_Y_14_ seeds were planted at 169 planting locations.

For the G_1_Y_13_ cohort, only a portion of fruit could be collected, because mature fruits dehisce explosively, and tulle bags used to cover fruit and thus retain seed were too heavy to apply to every fruit without damaging the plant. As newly initiated fruit appeared, the pedicel of each was marked with a paint pen for collection with a 0.5 probability. Fruits that were designated to disperse naturally were left unmarked. The marked fruits were later collected when mature while the unmarked fruits were permitted to dehisce naturally. This process resulted in sampling approximately 50% of all initiated fruits. By contrast, subsampling was not necessary for the G_1_Y_14_ and G_2_Y_14_ cohorts, because the stems were supported by 1‐m bamboo stakes so that they could withstand the weight of the tulle bags.

Because the goal of this experiment was to characterize the response to selection in a setting that closely matches a natural selective environment, seeds from collected fruit were hand‐planted adjacent to their maternal parent plant (Galloway & Etterson, 2007). Each hand‐planted seed was marked with a plastic toothpick to distinguish it from naturally dispersed seeds. G_2_Y_14_ seedlings derived from naturally dispersed seeds of G_1_Y_13_ were tracked within a 1.0‐m radius of the parental planting site, which includes the mean distance (57 cm) to which *C. fasciculata* seeds are known to disperse (Fenster, 1991). There was very low risk of confounding individuals arising from the naturally dispersed seeds with those arising from the seed bank: there was no evidence of *C. fasciculata* growing on‐site during visits in the previous growing season and *C. fasciculata* has low viability in the seed bank (Fenster, 1991). Data from naturally dispersed and manually planted G_2_ individuals were maintained separately prior to pooling in the final model (see Section 2.5). While it is possible that a small number of individuals tracked in the naturally dispersing group grew from seeds within the seed bank, the very few nonexperimental individuals observed in the vicinity of the experimental transects were destroyed. Families were not represented in equal proportions because families had differential reproductive output following hand pollination and natural outcrossing in the experimental garden (Table S2).

It is possible that the maternal environment experienced by the parents during the crossing efforts contributed to the observed phenotypic variation in this experiment. Two of the three crossing efforts took place in a greenhouse, where plants were relatively unstressed, while one crossing effort took place in a natural habitat, where individuals were more exposed to selection and to environmental variation. While the influence of the maternal environment cannot be eliminated, variance components and response to selection were estimated using paternal half‐sibling families, which are not expected to have a significant contribution from maternal effects (Falconer & Mackay, 1996).

### 2.4 Trait measurements

Evolutionary fitness was assessed by recording life‐history characters for individuals at each planting location (Figure 1c) throughout the 2013 and 2014 growing seasons. In addition to these measurements, we recorded two phenological traits: the ordinal dates of the first observed germinant and the first observed flower or fruit initiation at each planting location. In 2013, surveys for both germination and flower or fruit initiation were performed weekly, from 13 May (ordinal date: 132) until the first frost in October. In 2014, the large number of transects to be surveyed for germination and flowering necessitated a different procedure. For the 2014 trait measurements, starting on 12 May, each survey for phenological traits was performed over several days, as follows. On each day, a subset of transects from a randomized list was surveyed, and the number of new germinants or flowers or fruit initiated was recorded. Transects were randomized without regard to the cohort to eliminate bias against any year or generation. Recorded germination dates are not exact dates of first germination or initiated flowers or fruits, but rather the first observed germinant or initiated flower or fruit per planting location. The earliest observed germinant (hereafter EG) and earliest observed flower or fruit (hereafter EF) for each planting location were the phenological traits used to model fitness. *C. fasciculata* plants at the experimental site do not experience senescence. Rather, the first late‐summer or early‐fall frost date sets a hard end date on the reproductive period for the entire population. Thus, the earliest flowering individuals have the potential to have the longest reproductive period.

The interval between germination and flowering describes the developmental time required to transition between life‐history stages. This time can be summarized by the number of days from germination to flowering (DTF) and the total accumulated growing degree days from germination to flowering (GDD). Total accumulated GDD depends on both the number of days in the interval and the daily temperatures during the interval, with cool days contributing less to plant growth and development than warm days. Total accumulated GDD reflects not only the length of time to flowering, but also the development rate as a function of daily temperature. To calculate the total accumulated GDD, we used daily temperature data from the PRISM Climate Group (http://www.prism.oregonstate.edu), and a baseline temperature of 10 degrees Celsius, as is used for soybeans in Minnesota (Kandel & Akyuz, 2012).

### 2.5 Data analysis

Analyses were conducted using both a pedigreed dataset and the same dataset without parental information. Pedigree relationships were used to predict the response to selection, examine phenotypic plasticity, and estimate the additive genetic variance of germination and flowering times. Phenotypic plasticity was examined by a comparison of mean trait values of paternal families reared in two subsequent growing years (hereafter year‐environments). Additive genetic variance was estimated with the pedigreed dataset using the Quercus software package (Shaw & Shaw, 1994). Estimation of total lifetime fitness was performed with Aster models using the dataset not including pedigrees (Geyer et al., 2007). Selection gradients on EG and EF were estimated as *β*s according to a modified Lande and Arnold (1983) methodology as described by Geyer and Shaw (2010). The predicted response to selection was calculated using the additive genetic variance‐covariance matrix and the *β*s, according to Geyer and Shaw (2010). The observed response to selection in the second generation was evaluated as the differences in mean trait values between contemporary cohorts G_1_Y_14_ and G_2_Y_14_.

**Figure 2:**
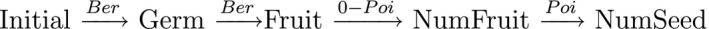
Graphical model of lifetime fitness. The nodes represent the number of seeds planted, whether or not a seed germinated, whether or not an individual survived to reproductive maturity, the number of fruit produced, and the number of seeds produced. The arrows denote dependence, and the labels denote the distribution associated with the dependence. Ber, Bernoulli; 0‐Poi, 0‐truncated Poisson; Poi, Poisson

Data were recorded for all experimental individuals according to their planting location; models used planting locations rather than individual plants. Total lifetime fitness and the relationships between fitness and EG and EF were estimated with fixed‐effects Aster models (Geyer et al., 2007; R Core Development Team, 2018). Aster models allow for the joint analysis of multiple components of lifetime fitness while accounting for both the dependency between life‐history stages and the distributional differences of life‐history stages (Geyer et al., 2007). An additional important property of Aster models is that “predecessor is sample size” (Geyer, 2013), meaning that nodes of the graphical model (Figure 2) are handled as sums of independent and identically distributed random variables. With this property, it is possible to model fitness of multiple individuals at single planting locations.

A graphical model describing the life‐history stages used in the Aster analysis is given in Figure 2. EG and EF were used as predictors of fitness in the analysis. Block was also included as a fixed effect to account for spatial environmental variation. In the model, the total number of seeds produced per seed planted at each planting location was used as the fitness measure (i.e., the number of seeds planted at a planting location was used as the “Initial” variable in the Aster model).

Mean fitness was estimated separately for each cohort. For G_2_Y_14_, data collected on both hand‐planted and naturally dispersed individuals were combined for analysis. We used the Aster package “predict.aster” function with predictor values set to block 4 and the observed values for EG and EF for each cohort. The “predict.aster” function estimates unconditional mean fitness for each planting location using an Aster model (Geyer et al., 2007). Block 4 was chosen for this because it is located at the experimental common garden midpoint, a convention used in previous studies that employed Aster modeling (Shaw, Geyer, Wagenius, Hangelbroek, & Etterson, 2008).

Development of the Aster model for this experiment began with a combined dataset that comprised all three cohorts. From this combined dataset, we performed backward model selection as described in Geyer and Shaw (2009) and Geyer (2013). The best‐fit model of the combined dataset was then used as a model selection starting point for each cohort. Final models for each cohort were chosen following the same methodology that was used for model selection with the combined dataset. For the initial G_1_Y_13_ model, two additional terms were tested for significance. The first was the effect of the two crossing efforts that produced this cohort (CrossType). The second term accounted for the inclusion of data belonging to individuals that germinated one year late (LateGerm); for G_1_Y_13_, this represents experimental individuals that grew in 2014 rather than 2013. For G_1_Y_14_, we also included the late germinants in the initial model.

Additive genetic variances for EG and EF were estimated with Quercus (Shaw & Shaw, 1994). Estimates of additive genetic variance for EG and EF used the dataset comprising individuals with known pedigree (G_1_Y_13_ and G_1_Y_14_). Data from 21 planting locations were excluded from the Quercus analyses because they did not have fully known pedigrees. The published version of Quercus analyzes only single generation data, so modification to the Quercus source code for analysis of multigenerational pedigrees was performed by F. Shaw (Shaw & Shaw, 1994). Modified Pascal code for Quercus was compiled with “fpc” version 3.0.2 and run on MacOS X 10.13.3. We initially ran Quercus without constraints, and then re‐ran it with constraints whether any variance estimates were negative. We also used the option to estimate common environmental variance rather than dominance variance for individuals that share parents. Block‐year combinations were treated as fixed factors in the model. Modified Quercus source code and a custom Python script to generate Quercus input files are available as Tables [Link], [Link].

We used fitness estimates from Aster models to estimate the strength of selection on EG and EF, following the methodology of Lande and Arnold (1983) and Geyer and Shaw (2010). While using fitness in ordinary least‐square regression analysis has been called into question (Mitchell‐Olds & Shaw, 1987), using estimates of fitness derived from Aster models alleviates issues related to the zero‐inflation of the fitness distribution. Absolute fitness estimates for each cohort were scaled to relative fitness values by dividing by the cohort's mean fitness. EG and EF were both centered about a mean of 0 by subtracting the cohort means from each observation. A multiple regression model was fit to each cohort using relative fitness as the response variable and centered values of EG and EF as phenotypic predictor variables. The regression coefficients (selection gradients, *β*s) were interpreted as strengths of directional selection on EG and EF. The *β*s and the additive genetic variance‐covariance matrix from Quercus were used to predict the response to selection for both EG and EF, following Geyer and Shaw (2010).

## 3 RESULTS

Of the 2,370 pedigreed first‐generation seeds comprising the Alpha G_1_Y_13_ cohort (Table 1), 29% were observed to germinate. The Beta cohort G_1_Y_14_ had much lower germination: only 7% of the 5,193 pedigreed seeds germinated. In the 2014 growing season, the germination rate of (G_1_Y_14_) was half that of its second‐generation contemporary (G_2_Y_14_: 14% of 6,842). G_1_Y_13_ had a per capita seed production greater than one seed per seed planted; G_1_Y_14_, on the other hand, produced proportionally fewer seeds (a reduction of nearly 19%). G_2_Y_14_ produced more seeds than the other 2014 cohort with 0.93 seeds produced per seed planted.

**Table 1:** Number of seeds planted, number of seeds germinated, number of seeds produced, and mean fitness estimates for each of the three cohorts in this study

	Alpha group		Beta group
	G_1_Y_13_	G_2_Y_14_	G_1_Y_14_
Seeds	2,370	6,842	5,193
Seeds germinated	694 (29%)	950 (14%)	378 (7%)
Seeds produced	7,088^a^ (25.3)	6,352 (157.2)	4,207 (18.5)
$\bar{W}$	3.39	2.93	7.55

a For G_1_Y_13_, the number of seeds produced accounts for the approximately 50% sampling fraction imposed on the fruits produced by this cohort. Numbers in parentheses for seeds produced are standard deviations of numbers of seeds from each planting location.

**Figure 3:**
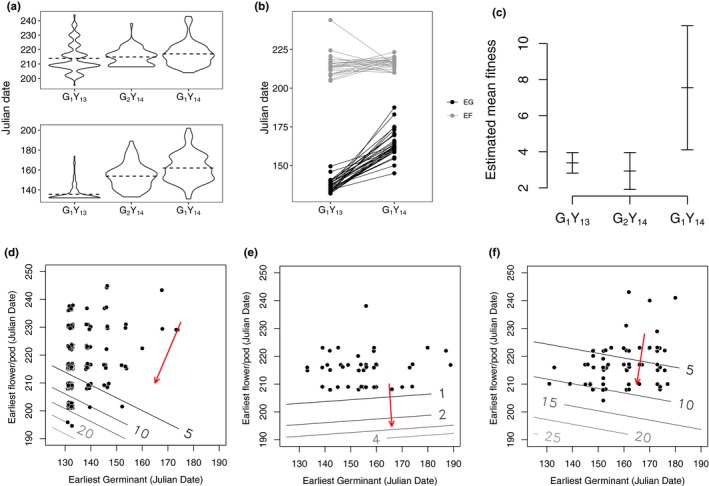
(a) Distributions of earliest observed germinant and earliest observed flower or fruit for each cohort. Horizontal dashed lines show means of the distributions. The distribution of EF for G_1_Y_13_ shows granularity because surveys for flower or fruit initiation were performed weekly in 2013. (b) First‐generation mean earliest observed flower or fruit initiated (EF) for paternal families, as grown in 2013 and 2014. The nonlinearity of the reaction norms indicates phenotypic plasticity for EG and EF in this population. (c) Mean fitness and standard error by cohort. Fitness estimates were derived from Aster models. (d) Fitness landscape of G_1_Y_13_. (e) Fitness landscape of G_2_Y_14_. (f) Fitness landscape of G_1_Y_14_. For d–f, points denote observed phenological values for a planting location, the lines show the contours of the fitness landscape, and the arrows show the direction of selection. The angle of the arrow indicates the selection gradients on EG and EF; the length of the arrow is arbitrary. Fitness landscapes were estimated following the methodology of Geyer and Shaw (2008b)

The distribution of EG for all cohorts varied considerably (Figure 3a). The average date of EG was considerably later in both 2014 cohorts (G_1_Y_14_ and G_2_Y_14_) than in the 2013 cohort (G_1_Y_13_). Standard deviations of EG were also greater in both 2014 cohorts. In 2013, 96% of G_1_Y_13_ individuals that would flower had germinated by the end of May; this is in contrast to 25% in G_1_Y_14_ and 41% in G_2_Y_14_, in which individuals that flowered had germinated over a wider range of dates and had a later mean germination date. We detected substantial additive genetic variation and narrow‐sense heritability for EG in pedigreed cohorts in this population (Table 4). To assess the effect of block, growth year (year‐environment), and generation on EG, we performed a series of ANOVA tests between pairs of linear models with and without each of these variables. Using a combined dataset including all three cohorts, the effect of year on EG was significant as tested by an ANOVA comparison of nested models. When comparing a combined model including all three cohorts, the effect of year was significant (*p* < .001). Using the same type of comparison, generation also had a significant effect on EG (*p* < .001).

The date of first observation of flowering or fruit initiation (EF) shows much higher phenotypic variability than EG. EF did not differ significantly between Alpha group generation G_1_Y_13_ and G_2_Y_14_ (*p* > .05). The bulk of observations for EF in both cohorts grown in 2014 (G_1_Y_14_ and G_2_Y_14_) were clustered around the mean, with a distinct tapering toward the end of the season (Figure 3a). Like EG, there is evidence of substantial additive genetic variance and heritability of EF in this population (Table 4). The estimates of *V*_A_ and *h* ^2^ were higher for EF than for EG. These two 2014 generations did not differ significantly with respect to mean EF (*p* > .05). Growth year did not have a significant effect on mean flowering date in the two G_1_ cohorts grown in different years. However, there is evidence of genetic variation in plasticity of flowering date in comparison of EF of G_1_ paternal families in 2013 and 2014 (Figure 3b). Further, growth year significantly contributes to variation in EF in G_1_ families in 2013 and 2014 (*p* < .001).

We also examined life‐history phenology using the interval between germination and flowering with two measures, total accumulated growing degree days from germination to flowering (GDD) and days to flowering (DTF). Average GDD and DTF were greater for both Alpha group cohorts (G_1_Y_13_ and G_2_Y_14_) than for the Beta cohort (G_1_Y_14_; Table 2). The contribution of year‐environment is apparent in the GDD and DTF values for the three cohorts: both G_1_Y_14_ and G_2_Y_14_ are more similar to each other with respect to mean developmental time than they are to G_1_Y_13_. However, because the traits of interest were the commencement of germination and flowering in this population, selection analyses were performed on EG and EF, rather than GDD or DTF.

**Table 2:** Cohort‐wide means and maternal family means of GDD and DTF for each cohort in this study

	Alpha group		Beta group
	G_1_Y_13_	G_2_Y_14_	G_1_Y_14_
Pop mean GDD	1,248.7 (146.9)	1,027.9 (198.3)	961.8 (182.8)
Mean Mat GDD	1,264.9 (117.7)	1,022.9 (162.2)	960.9 (186.8)
Pop mean DTF	79.3 (9.4)	61.4 (12.7)	56.6 (11.8)
Mean Mat DTF	79.8 (7.0)	61.2 (10.6)	56.4 (11.9)

Note

Means and standard deviations were calculated on a per planting location basis for cohort‐wide values and per‐family for maternal family values. Paternal family means and standard deviations are numerically similar to maternal means and standard deviations.

The first generation growing in the experimental garden in 2013 (G_1_Y_13_) had an estimated mean fitness ($\bar{W}$) of 3.77 seeds produced per seed planted (Table 1). This same value increased for G_1_Y_14_ to 7.55 and decreased for G_2_Y_14_ to 2.33. For each cohort, models that included only linear terms did not significantly differ from models with quadratic terms in regard to their residual sum of squares as assessed by a chi‐squared test of nested models (see Section 2).

In all cohorts, both types of selection gradients for EF (Lande‐Arnold βs and the Aster model fitness:EF regression coefficient) were significantly less than zero, indicating selection for earlier flowering. The effect of crossing effort (CrossType) was not significant in the model. The estimated fitness landscape for G_1_Y_13_ (Figure 3d) shows that fitness increased with earlier EG and EF; the regression coefficients for each were both significantly less than zero. Selection was stronger on EF than on EG in G_1_Y_13_ (Table 3). The fitness landscape for both Alpha cohorts (G_1_Y_13_ and G_2_Y_14_) show similar patterns, but the magnitude of estimated fitness is much lower for G_2_Y_14_ (Figure 3e). While it appears that there was selection for later EG in G_1_Y_14_, the regression coefficient for EG in the model is not significantly different from zero. The fitness landscape of G_1_Y_14_ is qualitatively similar to that of G_1_Y_13_ (Figure 3f).

**Table 3:** Estimates of selection gradients on EF and EG derived from Lande and Arnold OLS regression analysis (*β*) and Aster models (fit:trait)

	Alpha group		Beta group
	G_1_Y_13_	G_2_Y_14_	G_1_Y_14_
*β*_EG_	−0.0124	0.0062	−0.0115
*β*_EF_	−0.0623***	−0.0900**	−0.0385*
fit:EG	−0.0066*	0.0003	−0.003
fit:EF	−0.0144***	−0.0048***	−0.0018**

Note

Stars denote significance: *p < .05; **p < .01; ***p < .001.

Both types of selection gradients for EF were significantly different from zero and in the same direction in all cohorts. By contrast, the only statistically significant selection gradient for EG was the Aster‐derived gradient for G_1_Y_13_. Using the additive genetic variance‐covariance matrix estimated by Quercus, the response to selection predicted for G_2_Y_14_ by Lande and Arnold regression on G_1_Y_13_ was advancement of EG by 0.8 days and advancement of EF by 1.1 days (Table 5). For both EG and EF in all three cohorts, the selection gradients from Lande and Arnold analysis had a larger magnitude than those from the corresponding Aster models. This is likely due to bias in the predicted fitness values from the ordinary least squares regression used by the Lande–Arnold method. These regression models for each cohort have residuals which are centered above zero, indicating a systematic overestimation of fitness conditional on EG and EF values.

## 4 DISCUSSION

Observed onset of germination differed between generations and growing years, while mean flowering time only differed between years. In this population, differences in life‐history phenology were clearest in comparisons between year‐environments, as opposed to comparisons between generations. When first and second generations were grown in the same year (G_1_Y_14_ and G_2_Y_14_) the observed advancement of mean flowering time by 2.2 days was consistent with the direction of selection predicted by the Aster model of its parent generation (G_1_Y_13_) in 2013.

### 4.1 Phenological differences between years

The dependence of germination phenology on environmental conditions has been observed in previous studies. For example, a study by Donohue et al. (2005) found plasticity for germination onset in *Arabidopsis thaliana* in field conditions at two sites. They found that site by genotype interactions contributed significantly to variation in germination time. Additionally, germination at the cooler of their field sites (Rhode Island) was found to occur later than at the warmer field site (Kentucky). Donohue et al. (2005) demonstrate phenotypic plasticity for germination onset along a spatial environmental gradient, while our study examines plasticity along a temporal‐environmental gradient. Although spatial and temporal effects are not equivalent, successive year‐environments similarly represent distinct growth‐environments. Based on the results of previous studies (e.g., Fernández‐Pascual & Jiménez‐Alfaro, 2014), we speculate that interannual variation in temperature was a major contributor to plasticity in our study. While other environmental factors, such as burning or mowing may have contributed to differences in germination time, delayed germination of the 2014 cohorts coincided with the substantially colder temperatures of the 2013–2014 winter. Specifically, Shakopee, Minnesota winter temperatures in 2013–2014 were much colder than in 2012–2013 winter (Table S3), corresponding with delayed germination dates in the latter year.

In the current study, we also observed evidence phenotypic plasticity of EG. Among the paternal families reared in two different growth years, some families were relatively stable across years, while others were observed to germinate late in 2013 but relatively early in 2014 (Figure 3b). While such plasticity may be beneficial in the short term, it does not contribute to a long‐term response to selection (Duputié, Rutschmann, Ronce, & Chuine, 2015).

Another way to explore the life‐history phenology in this study is by examining the interval between germination and flowering using GDD and DTF. The average DTF in G_1_Y_14_ was shorter than in G_1_Y_13_, a result that is likely due to later mean germination in G_1_Y_14_ (Table 2). This shortened time to maturity is also reflected when accounting for the contribution of daily temperatures to plant development, as is shown by a lower average GDD at the study site in 2014 than in 2013. It appears that in this population, delaying germination need not delay reproduction. This insensitivity of flowering date has been observed in previous studies, but variation in flowering date appears to be species‐specific. Lu, Tan, Baskin, and Baskin (2016) observed no effect of germination date on time to maturity in a facultative winter annual, while Zhou, Wang, and Valentine (2005) found that sowing date affects the time to maturity in two spring annuals. Furthermore, Zhou et al. (2005) also reported that plants with later sowing dates develop more rapidly than plants with earlier sowing dates. Our findings suggest that this experimental population of *C. fasciculata* responded similarly to the pattern described by Zhou et al. (2005), rather than the pattern reported by (Lu et al., 2016). Rapid development of late germinants in our study may have contributed to the observed stability of the phenotypic distribution of EF between years.

### 4.2 Selection on phenology in each cohort

In the 2013 G_1_ cohort, selection favored both earlier germination and earlier flowering. A significant selection gradient on EG is not seen in their offspring in 2014 (G_2_Y_14_). While this gradient is not significantly different from zero in G_2_Y_14_, it appears to switch directions compared to G_1_Y_13_, with selection for earlier germination changing to selection for later germination. Additional observations of paired first and second‐generation cohorts may better reveal the nature of selection, as longer‐term studies may better account for the effect of year‐to‐year environmental fluctuations.

Maternal effects, both due to genetic and correlated environmental factors, are important components of selection in natural populations (Galloway & Etterson, 2007). The correlation between microenvironmental conditions of the maternal parent and offspring in natural populations was included in our study by planting offspring adjacent to their maternal parents. The experimental planting locations around each maternal parent were encompassed by the seed dispersal distance of *C. fasciculata* of approximately 57 cm (Fenster, 1991).

Aster selection gradients for all three cohorts indicate that earlier‐flowering plants have higher fitness than late‐flowering plants (i.e., there is significant directional selection for earlier flowering). This is similar to findings in other species (Ehrlén & Münzbergová, 2009; Franks, Sim, & Weis, 2007). Given that there was selection for earlier flowering, the second generation of our study would be expected to have an earlier mean flowering date than the first generation, assuming that the first generation harbored sufficient genetic variation in flowering date to support a response to selection. Comparing parental (G_1_Y_13_) to progeny (G_2_Y_14_) values, mean flowering time is later in the growing season following one generation of selection. While this appears to contradict the significant directional selection for earlier flowering, the response is clear when considering the effect of the environment. Growing the first and second generation cohorts in the same year allows us to control for the effect of the environment, and more clearly observe the response to selection. In accordance with phenotypic selection analysis, when first (G_1_Y_14_) and second (G_2_Y_14_) generations are grown concurrently, a potential response to selection can be seen. The mean flowering time of G_2_Y_14_ advanced 2.2 days with respect to its first‐generation contemporary cohort (G_1_Y_14_)_._

Selection gradients as estimated by ordinary least squares (OLS) regression were qualitatively consistent with those obtained from Aster models. The direction of selection was consistent, and the strength of selection inferred by OLS regression was much stronger than selection gradients produced by Aster models (Table 3). Aster models appropriately model the zero‐inflation of the distribution of fitness, which removes the upward bias observed in analyses that do not account for it (Geyer et al., 2007). Despite using fitness estimates derived from Aster models, the residuals of the OLS regression are not centered on zero, showing bias in the estimation of fitness conditional on germination and flowering time. Pearson residuals from the Aster models do not show such bias. Further, OLS regression to estimate the relationship between fitness and phenotypic predictor traits relies on the assumption that the phenotypic predictors are multivariate normal, which is often violated in empirical datasets including our own (Figure 3a; Geyer & Shaw, 2008a).

### 4.3 The effect of the environment

The data presented in this study allow for three ways to assess the effect of the environment on phenotypic variation. The first is estimation of heritability in multiple environments. The second way is comparing relative trait values of pedigreed families in multiple environments to observe phenotypic plasticity. The final way is to by measurement of the selective environment over multiple years.

The respective contributions of genetic and environmental effects can be compared by estimating the heritability of a trait, that is, *h* ^2^ = *V*_A_/*V*_P_ (Falconer & Mackay, 1996). Because the environmental variance is defined as the portion of phenotypic variance that is not due to genetic effects, heritability is a way to compare the relative contribution of the environment to phenotypic variation. The heritabilities of EG and EF show that environmental variance was the primary contributor to phenotypic variance in this population (Table 4).

**Table 4:** Estimates of additive genetic variance, environmental variance, narrow‐sense heritability, additive genetic correlation, and environmental correlation for EG and EF in this study population

	EG	EF
*V*_A_	3.84	15.71
*V*_E_	121.9	75.24
*h* ^2^	0.030	0.173
Cor_A_	1.36	
Cor_E_	0.40	

Note

All estimates were based on G_1_ individuals with resolved pedigree information, which includes G_1_Y_13_ and G_1_Y_14_.

Abbreviations: Cor_A_, additive genetic correlation; Cor_E_, environmental correlation.

Another way to assess the effect of the environment is by evaluating a population for interaction effects of genotypes (pedigreed families) and trait values across multiple growing environments. Growth year did significantly contribute to variation in EF among all cohorts in this study (*p* < .001). A change in the rank order of the mean phenotypes of families across multiple environments indicates phenotypic plasticity in the recorded trait. Paternal half‐sibling family trait means in G_1_Y_13_ and G_1_Y_14_ are suggestive of phenotypic plasticity (Figure 3b). While the change in rank order of paternal families does not necessarily quantify the magnitude of environmental effect, it provides insight into how the environment affects the expression of genetic variation.

### 4.4 Relationships between the phenology of individual life‐history traits and fitness

Artificial selection experiments using plants (e.g., Burgess, Etterson, & Galloway, 2007; Galloway & Burgess, 2012) and observational studies in vertebrates (e.g., Réale et al., 2003) have demonstrated genetically based responses to selection on life‐history phenology. In this study, we identify a genetically based response to selection by separating the effect of the environment from the total phenotypic change between generations. It is important to note that while the selection gradient was significant, the difference in mean trait values was not.

Analysis of the genetic variance components for phenological traits showed substantial additive genetic variance for EF, but not EG. Despite selection being significant on only one of the phenological variables, the additive genetic correlation between EF and EG is >1, which implies a very strong genetic correlation between germination date and flowering date (Table 4). This may explain the statistically nonsignificant selection gradient estimates for EG. If traits used to predict fitness are highly correlated with each other, then the power to detect selection in a joint analysis with those traits can be very low (Shaw & Geyer, 2010).

The observed positive genetic correlation between EG and EF has several potential contributing factors. One is pleiotropy, where the genetic loci that contribute to variation in germination time also contribute to variation in flowering time. Another possibility is that the transition to flowering is triggered after a certain amount of accumulated GDD or DTF. In this case, the interval between germination and flowering would be the trait under selection; measurements of EG or EF would then be measurements of the same trait and, thus, be highly correlated. Finally, because the source populations for the experimental individuals are near the northern range edge, they may have high linkage disequilibrium among genetic loci that contribute to variation in germination time and loci that contribute to variation in flowering time due to local inbreeding (reviewed in Loveless & Hamrick, 1984).

When explicitly measuring the contribution of life‐history phenology to fitness variation, we find different patterns for flowering date and germination date. Similar to a previous study in *Brassica rapa* (Austen & Weis, 2015), we find a stable relationship between fitness and flowering date, with earlier flowering resulting in higher fitness. The relationship between germination date and fitness is more complex. While previous studies have found a large contribution of germination phenology to fitness variation (Donohue et al., 2005), we did not observe a consistent relationship in our data. Instead, we observe a significant contribution of germination phenology to fitness in the 2013 cohort, but not in the 2014 cohorts, which is similar to the findings reported by Kalisz (1986).

Comparisons of flowering time within the Alpha group (G_1_Y_13_ and G_2_Y_14_) conflate the effects of selection with the contribution from variation in environmental conditions. The onset of flowering was predicted to advance by 1.1 days in G_2_Y_14_ relative to G_1_Y_13,_ but the observed change was a delay of 0.9 days (Table 5). When comparing between Alpha and Beta cohorts, such as G_1_Y_14_ and G_2_Y_14_, we can better isolate the effects of selection, because both generations were reared in the same year‐environment. Comparisons between Alpha and Beta cohorts show a change in mean flowering time consistent with the direction of selection on EF. Further, the G_2_ cohort shows a contraction of the distribution of EF compared to both G_1_ cohorts (Figure 3a, Table 5), which is one of the expected outcomes of directional selection on EF (Falconer & Mackay, 1996).

**Table 5:** Observed means of EG and EF in each cohort and their predicted means in response to selection in unobserved G_2_ cohorts

	Alpha group			Beta group	
	Obs	Pred	Obs	Obs	Pred
	G_1_Y_13_	G_2_Y_14_	G_2_Y_14_	G_1_Y_14_	G_2_Y_15_
Mean EG	135.7 (8.2)	134.9	153.8 (12.7)	162.0 (14.2)	161.5
Mean EF	213.9 (9.8)	212.8	214.8 (5.9)	217.0 (8.1)	216.2

Note

Predictions were generated with the additive genetic variance‐covariance matrix from Quercus and the Lande–Arnold selection gradients. Numbers in parentheses are standard deviations on a planting location basis.

When comparing the fitness landscapes of all three cohorts, the estimates of total lifetime fitness (absolute fitness) in the G_2_ cohort are 10‐fold lower than those in the G_1_ cohorts. These differences may arise from several sources. First, the association between germination and fitness is complex; individuals that germinate earlier face less competition from neighbors but may also experience more intense herbivory (Stanton‐Geddes, Tiffin, & Shaw, 2012). Since the estimates of fitness from Aster models incorporate viability and fecundity, the magnitudes of the fitness estimates may depend on germination time. Second, the selective environment is known to differ from year to year (Ollerton & Lack, 1998), and the environment is known to be the largest contributor to phenotypic variation in natural populations (Falconer & Mackay, 1996). We have detected directional selection for earlier flowering in all cohorts, though the intensity of selection on flowering date differs between years. The effect of growth year was a significant source of variation in both life‐history phenology and absolute fitness. Interactions between selective environment, genetic variances and correlations of traits, and life‐history phenology have complex impacts on variation in absolute fitness.

## CONFLICT OF INTEREST

None declared.

## AUTHORS CONTRIBUTIONS

A. R. N. involved in study design, crossing, experimental garden planting, and data collection. A. R. N. and T. J. Y. K. involved in data analysis and writing.

## Data Availability

All data analyzed for this study are deposited into Dryad and are available at https://doi.org/10.5061/dryad.gqnk98shj.
