# Supplementary material for: Selection and plasticity both account for interannual variation in life‐history phenology in an annual prairie legume
Source: Ecol Evol. 2020 Jan 10;10(2):940–51. doi: 10.1002/ece3.5953 (PMC6988531; doi:10.1002/ece3.5953)
Supplement: Supplementary file 1 [file ECE3-10-940-s001.docx]

| Pedigreed Cohort | Group | Crossing Design | Crossing Year | Crossing Location |
| --- | --- | --- | --- | --- |
| G_1_Y_13_ | Alpha | Reciprocal Factorial | 2012 | Greenhouse |
|  |  | Paternal Nested | 2012 | Field |
| G_1_Y_14_ | Beta | Reciprocal Factorial | 2013 | Greenhouse |

Table S1: The three crossing efforts, represented as rows, which generated the G_1_ cohorts in this study.

|  | Alpha Group | | Beta Group |
| --- | --- | --- | --- |
|  | G_1_Y_13_ | G_2_Y_14_ | G_1_Y_14_ |
| Number of Paternal Families | 77 | --- | 138 |
| Number of Maternal Families | 111 | 63 | 121 |
| Mean Dams Per Sire | 2.05 | --- | 3.08 |
| Mean Seeds Planted Per Dam | 20.54 | 37.21 | 42.92 |
| Mean Seeds Planted Per Sire | 29.61 | --- | 37.63 |

Table S2: Summary of the crossing structures and representation of families in each cohort in this study.

Caption

Table S3 Mean temperatures for winter months at the Shakopee study site. Prism 2016
