# Supplementary material for: Selection and plasticity both account for interannual variation in life‐history phenology in an annual prairie legume
Source: Ecol Evol. 2020 Jan 10;10(2):940–51. doi: 10.1002/ece3.5953 (PMC6988531; doi:10.1002/ece3.5953)
Supplement: Supplementary file 2 [file ECE3-10-940-s002.pdf]

	December	January	February
2012-2013	-5.0°	-8.4°	-8.1°
2013-2014	-11.0°	-13.6°	-13.6°
30-Yr Normal	-6.0°	-9.1°	-6.5°
